# Effects of Synergism of Mindfulness Practice Associated With Transcranial Direct-Current Stimulation in Chronic Migraine: Pilot, Randomized, Controlled, Double-Blind Clinical Trial

**DOI:** 10.3389/fnhum.2021.769619

**Published:** 2021-12-08

**Authors:** Luana Dias Santiago Pimenta, Elidianne Layanne Medeiros de Araújo, Joyce Poláine dos Santos Silva, Jamyson Júnior França, Pedro Nascimento Araújo Brito, Ledycnarf Januário de Holanda, Ana Raquel Lindquist, Luiz Carlos Serramo Lopez, Suellen Marinho Andrade

**Affiliations:** ^1^Graduate Program in Neuroscience and Behavior, Federal University of Paraíba, João Pessoa, Brazil; ^2^Physiotherapy, Federal University of Paraíba, João Pessoa, Brazil; ^3^Medical, Federal University of Paraíba, João Pessoa, Brazil; ^4^Graduate Program in Physical Therapy, Federal University of Rio Grande do Norte, Natal, Brazil

**Keywords:** transcranial direct-current stimulation, mindfulness, full attention, chronic migraine, electrostimulation

## Abstract

Chronic migraine is a difficult disease to diagnose, and its pathophysiology remains undefined. Its symptoms affect the quality of life and daily living tasks of the affected person, leading to momentary disability. This is a pilot, randomized, controlled, double-blind clinical trial study with female patients between 18 and 65 years old with chronic migraine. The patients underwent twelve mindfulness sessions paired with anodal transcranial direct-current stimulation (tDCS) over the left dorsolateral prefrontal cortex (DLPFC), with current intensity of 2 mA applied for 20 min, three times a week for 4 weeks. In addition, 20 min of mindfulness home practices were performed by guided meditation audio files. A total of 30 participants were evaluated after the treatment, and these were subdivided into two groups—active tDCS and sham tDCS, both set to mindfulness practice. The FFMQ-BR (Five Facet of Mindfulness Questionnaire), MIDAS (Migraine Disability Assessment), and HIT-6 (Headache Impact Test) questionnaires were used to evaluate the outcomes. After the treatment, the active mindfulness and tDCS group showed better results in all outcomes. The sham group also showed improvements, but with smaller effect sizes compared to the active group. The only significant difference in the intergroup analysis was the outcome evaluated by HIT-6 in the post treatment result. Our results provide the first therapeutic evidence of mindfulness practices associated with left DLPFC anodal tDCS with a consequent increase in the level of full attention and analgesic benefits in the clinical symptoms of patients with chronic migraine.

## 1. Introduction

Chronic migraine is a primary multifactorial neurological disorder (Vos et al., 2016, 2017; James et al., 2018) which is common and incapacitating. It is defined by the occurrence of headache on 15 days or more per month, with at least 8 of these days having the characteristics of migraine, for more than 3 months (Arnold, 2018). It has an estimated prevalence of 13–18% of the world population and its pathophysiology seems to be more linked to abnormalities in the neural network of pain resulting from morphofunctional alterations in brain regions such as the dorsolateral prefrontal cortex (DLPFC), mesencephalic structures and the cingulate cortex (Marcus, 2003; Lipton et al., 2008). The increase in the frequency of the crises inherent to the pathology leads to a greater central sensitization, predisposing the sufferer to a “vicious cycle of pain” (Burstein, 2001; Chiapparini et al., 2010) and further predisposing them to potential negative repercussions in the biopsychosocial sphere in the face of the frequent association with absenteeism from work, anxiety, depression, sleep disorders and decreased socialization (Mercante et al., 2005; Stuginski-Barbosa and Speciali, 2011).

Therapeutic prophylaxis is strongly recommended in patients with severe and/or frequent impairment related to headache cephalalgy. The main treatment is still pharmacological, although most drugs are not very specific, and can trigger side effects which are not tolerable, and their excessive consumption predisposes the user to a diagnostic association with migraine due to the abusive use of drugs (Goadsby and Sprenger, 2010; Shukla and Sinh, 2010; Buse et al., 2012; Parra et al., 2015; Rocha et al., 2015; Shirahige et al., 2016). In this context, non-pharmacological therapies have been shown to be allied to lower drug consumption and frequency of headaches, with greater acceptance and safety compared to current therapy (Peres et al., 2011).

Mindfulness, whose core was possibly originated in meditative practices of ancient Taoism, Hinduism, and Buddhism, is the consciousness of intentional and non-judgmental attention to the present moment. Its exercise enables people to focus on present experiences, interrupting the trend of daily automation, as well as providing the cultivation of non-judging attitudes, emotions and pain (Andrasik et al., 2016). There have been substantial advances in the knowledge of the neural mechanisms related to mindfulness practices. Electroencephalographic (EEG) and functional magnetic resonance imaging (fMRI) studies suggest that mindfulness exercise induces changes in the brain “state,” including activations of the anterior cingulate cortex and the dorsomedial prefrontal cortex (Davidson et al., 2003; Hölzel et al., 2008; McCallion, 2017). Mindfulness-based interventions can produce similar effects to isolated medication for chronic migraine patients with a history of too much pharmacological use (Grazzi et al., 2017).

Thus, Mindfulness-based therapeutic interventions have been used as a single or co-adjuvant treatment for migraines (Day et al., 2014). This technique provides greater attentional and emotional self-regulation and self-perception (Gu et al., 2018), generating physical, mental and social well-being benefits, in addition to minimizing symptoms related to anxiety, stress, depression, and rumination, which are considered triggers for migraine attacks (Badran et al., 2017). Previous neuroimaging studies comparing experienced meditators to non-meditators point to the existence of a greater volume of the gray substance in the hippocampal and frontal cortex in regular practitioners (Luders et al., 2009; Chételat et al., 2017), which is also related to long-term changes in hippocampus functional topology (Lardone et al., 2018).

Transcranial direct-current stimulation (tDCS) is a neuromodulatory technique that enables greater pain control and a decrease in the clinical symptoms of migraine, as it can modulate the excitability and excessive cortical neural hyperresponsiveness inherent to this pathology and can be used, both in prophylaxis and in crisis intervention (Machado et al., 2009; Peres et al., 2011; Magis, 2015; Parra et al., 2015; Shirahige et al., 2016).

Studies have shown that the combination of mindfulness practice and tDCS seems to have a synergistic effect in reducing osteoarthritis pain (Ahn et al., 2019), in improving the working memory in individuals without neurological comorbidities (Hunter et al., 2018) and the positive affective experience in university students (Robinson et al., 2019). For example, improvements in parameters such as attentional inhibition, cognition and executive function skills were observed in a study associating mindfulness practice and tDCS of the left DLPFC of patients with refractory depression compared to conventional pharmacological treatment (Monnart et al., 2019). A recent study demonstrated that tDCS can improve mindfulness skills learning by an intervention program based on this technique in a group of adults with history of chronic pain (McCallion et al., 2020).

In view of the above, this study aimed to associate mindfulness practice with left DLPFC anodal tDCS as a prophylactic synergistic therapy in painful symptoms of patients diagnosed with chronic migraine. Specifically, we aim to increase the level of full attention of these patients with their potential benefits in order to minimize pain, decrease the degree of inability to perform activities of daily living and the negative impact of this clinical condition on patients' daily lives.

## 2. Materials and Methods

This study was approved by the Institution's Ethics Committee, conducted in accordance with the 1964 Helsinki Declaration and registered on the Clinical Trials platform (www.clinicaltrials.org) (NCT04219345). Written and informed consent was obtained from all participants.

### 2.1. Study Design

This is a pilot, parallel, placebo-controlled, double-blind, randomized clinical trial in accordance with the Consolidated Standards of Reporting Trials (CONSORT) guidelines (Moher et al., 2012). The study was conducted in a Public Neuromodulation Unit, which provides assistance specialized to patients with neurological and psychiatric disorders.

Patients were interviewed during the first visit for clinical migraine diagnosis and assessment of eligibility criteria. At this time, the participants were instructed on how to complete the registration document of home mindfulness practices. This record served to observe whether home practices were taking place daily and without interruptions.

### 2.2. Participants

Female patients aged between 18 and 65 years who had at least 1 year of confirmed diagnosis of chronic migraine according to the International Classification of Headache Disorders (ICHD-3 beta) (International Headache Society, 2016) of the International Headache Society were included. We selected patients receiving stable doses of pain medication for at least 2 months before the start of this study. Exclusion criteria were patients with headache attributable to another associated neurological or neuropsychiatric disease, using central nervous system (CNS) modulating drugs, undergoing other non-drug therapy for migraine or other CNS pathologies concurrent to the intervention period or 2 months prior to this, being pregnant, presenting a metallic implant located in the cephalic region or with a cardiac pacemaker.

### 2.3. Randomization and Blinding

Participants were randomly allocated in a 1:1 ratio to receive tDCS sham or anodic active tDCS in the left DLPFC region, both paired with mindfulness through an online generator (www.random.org). The hidden allocation process was carried out using sequential, numbered, opaque and sealed envelopes. Outcome evaluators, subjects and patients were blinded to the procedures performed.

We asked participants at the endpoint to guess which group they were allocated to in order to assess the effectiveness of blinding, and rated the confidence of their assumption on a Likert scale (Poreisz et al., 2007).

### 2.4. Outcomes

The primary outcome was the performance of the FFMQ-BR questionnaire (Five Facets of Mindfulness Questionnaire), an instrument which assesses variations in mindfulness characteristics, subdivided into five main factors (observing, describing, not judging, not reacting to experiences and acting) to assess the participants' mindfulness level (Barros et al., 2014).

One of the secondary outcomes was assessed by the MIDAS questionnaire (Migraine Disability Assessment Questionnaire), which is an instrument that assesses the inability to perform daily and professional tasks in patients with migraine and that can be applied to people with different educational levels and social backgrounds due to the fact that it is considered easy to be answered (Fragoso, 2002). This instrument contains five questions that were filled with the number of days on which the patient stopped performing the specified activity because they were experiencing a migraine episode, which is a useful tool to identify the severity of the pathology. The other secondary outcome was analyzed by the results of the Headache Impact Test-6 (HIT-6), a questionnaire that assesses the frequency of headache impact on migraine patients' quality of life (social aspects, functional role, vitality, functioning and psychological suffering), considered easy to apply and having a high reliability index (Yang et al., 2011).

### 2.5. Intervention

**Figure 1:**
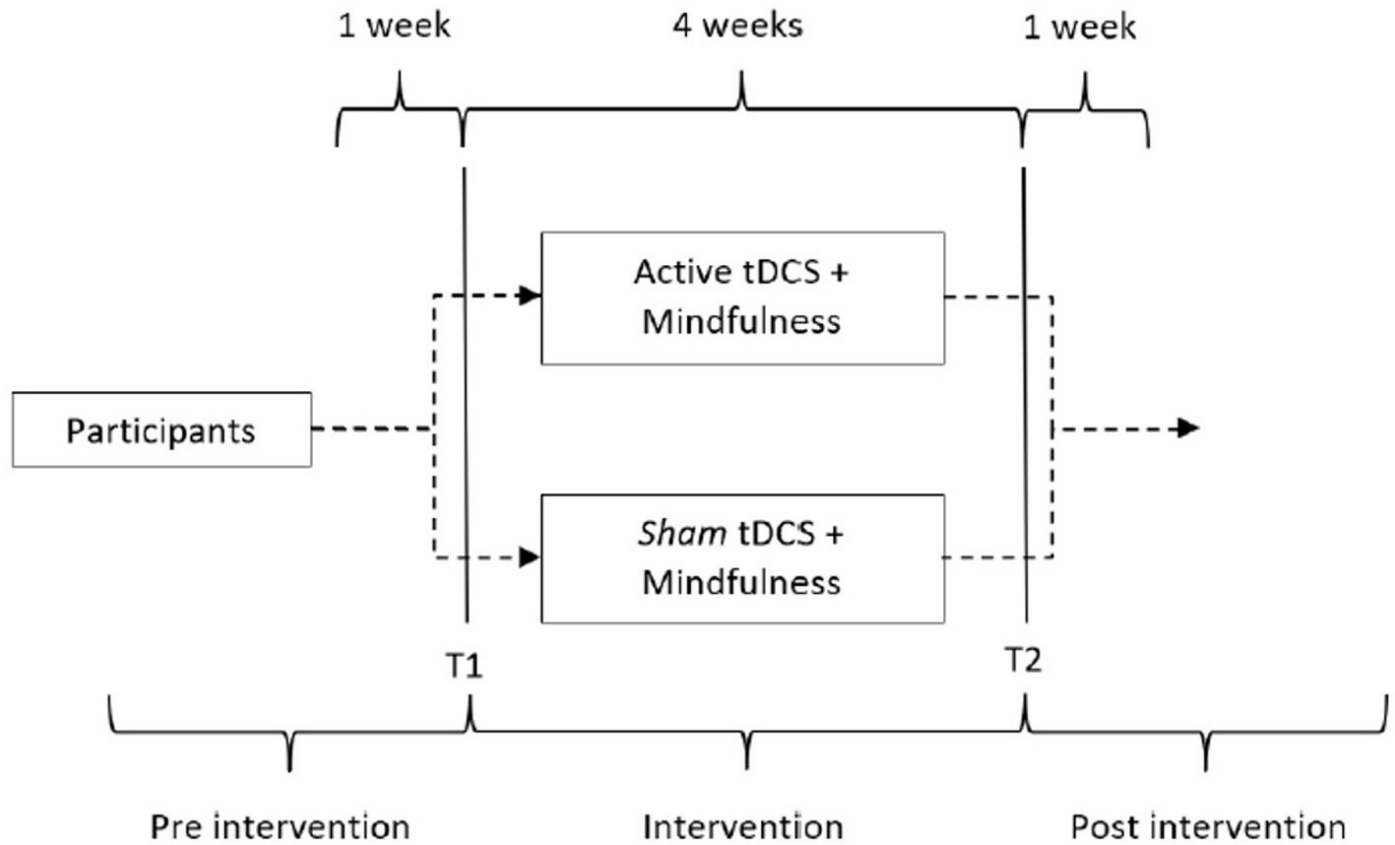
Active tDCS study design vs. sham, both associated with mindfulness in chronic migraine. T1, baseline; T2, end of intervention.

The patients were submitted to 12 treatment sessions, distributed three times a week, for 4 weeks (Figure 1). The direct current was transferred by means of a TransCranial Technologies neurostimulator (Hong Kong, China), using electrodes and 5x5 cm sponges moistened with a saline solution (0.9% sodium chloride). The electrodes were positioned in accordance with the 10-20 EEG landmarks. The 2.0 mA intensity current was applied for 20 min by anodic stimulation in the left DLPFC (position F3). The reference electrode was placed over the right supraorbital region (Fp2 position) (DaSilva et al., 2015). The target regions were located by an experienced and trained professional. The electrodes were positioned in the same setting for the sham stimulation, but the current was automatically turned off after 30 s. The participants sat in a comfortable chair in a noise-free environment during the procedure.

At the end of the session, each patient was asked if they felt any adverse effects such as dizziness, tingling, burning, headache, somnolence, and others and the intensity of this sensation (1—no sensation, 2—mild, 3—moderate, 4—intense).

**Figure 2:**
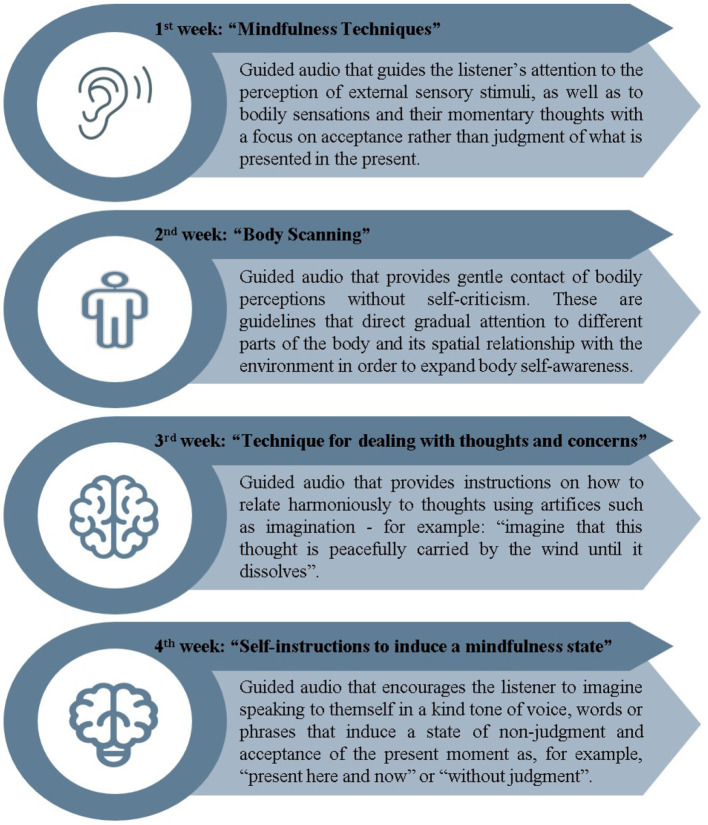
Mindfulness protocol used in the 4 weeks of treatment.

The mindfulness practices were performed by listening to guided audio recorded by an experienced and specialized instructor, and were concurrently carried out with the tDCS application. The audios were later made available via email or WhatsApp, so that the participants could perform alone on days when the associated therapy did not occur. A different audio content was provided each week, totaling 28 days of mindfulness exercises, 12 of which were practiced together with tDCS. The subjects were requested to register the practices on an individual table for monitoring adherence to home exercises. The systematization and theoretical basis of the audio content was chosen from previous studies involving guided mindfulness application (Azam et al., 2016; Howarth et al., 2019). The protocol used in the 4 weeks of treatment is briefly described below (Figure 2).

### 2.6. Statistical Analysis

All reviews were performed in the Statistical Package for the Social Sciences version 25.0, with a significance level of 5%. The statistics were performed based on the intent to treat (ITT) analysis. The sample size was estimated using data from our previous study with tDCS (Andrade et al., 2015) and from studies involving mindfulness combined with tDCS in pain patients (Ahn et al., 2019; Witkiewitz et al., 2019). Thus, considering a paired difference of 1 or more in MIDAS to be significant with a power of 80% and an alpha of 5%, with a dropout rate of 10%, the minimum number of patients needed was estimated at 30.

The descriptive analysis was performed using mean, standard deviation, and frequency. The Shapiro−Wilk test was used to check normality and, as not all measurements were normally distributed, the Mann−Whitney test was used for all outcomes in the intergroup comparison (group, active tDCS and mindfulness vs. sham tDCS and mindfulness) and the Wilcoxon test for the intragroup comparison (time, baseline vs. endpoint). Finally, the occurrence of adverse events was performed with the corresponding descriptive analysis of each event.

*P*-values were adjusted by the Bonferroni method in all statistical analyses, a *p*-value of < 0.05 was considered significant and the Pearson's effect size “*r*” was considered as: *r* ≤ 0.10 small, *r* = 0.30 medium, and *r* ≥ 0.50 large.

## 3. Results

### 3.1. Participants

**Figure 3:**
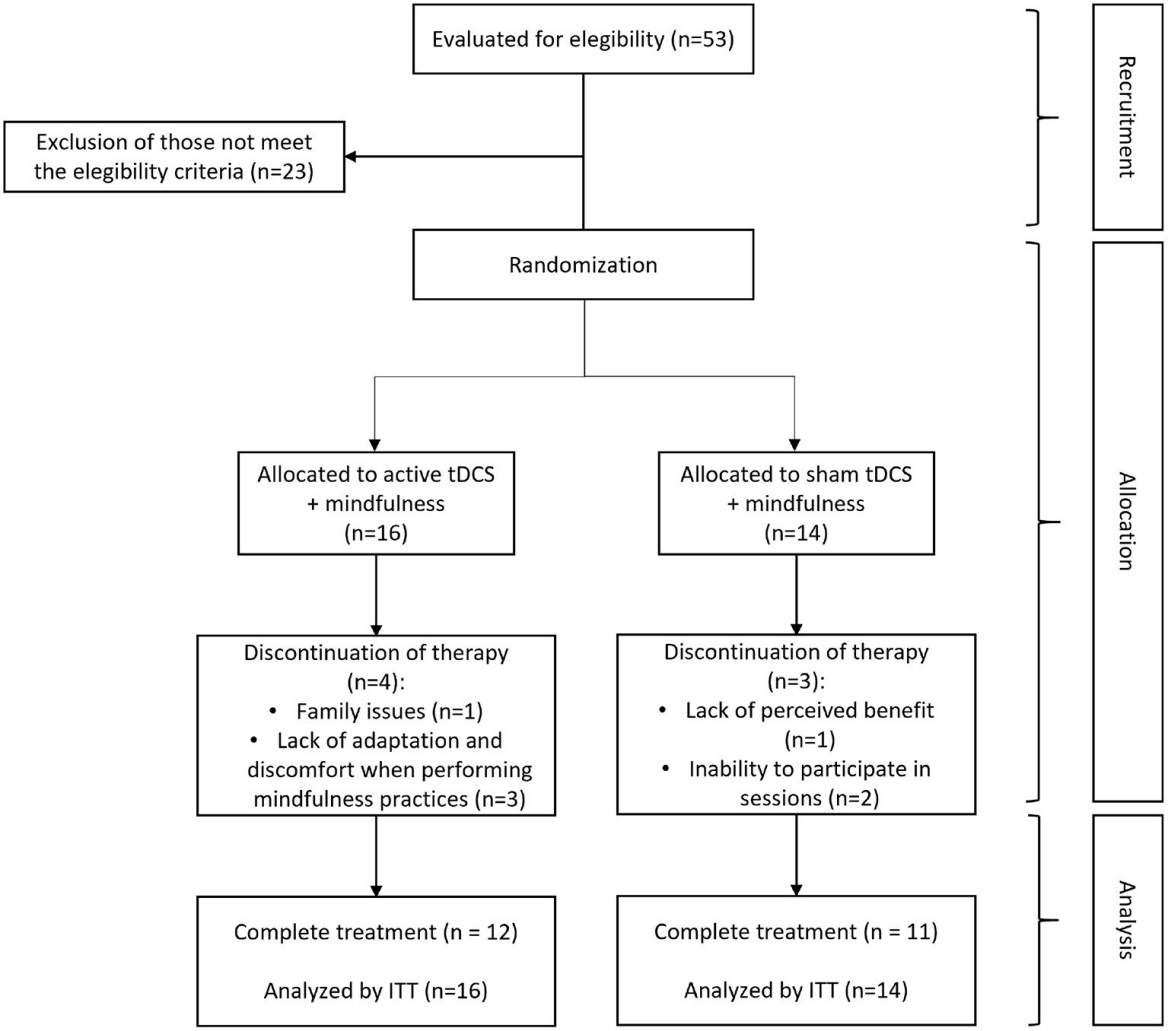
CONSORT flowchart. *n*, quantity of participants; ITT, intention to treat.

A total of 53 patients with chronic migraine were screened, 30 of whom were eligible to participate in the study and were randomly allocated to both groups. In total, four patients left the study after receiving passive stimulation and three after receiving active stimulation. The reasons for leaving the active group were family problems (one participant) and lack of adaptation and discomfort when performing mindfulness practices (three participants). The reasons in the sham stimulation group were the lack of perceived benefits (one participant) and impossibility to participate in the sessions (two participants). In total, 23 patients successfully completed the study (Figure 3). At the end of the twelve joint intervention sessions of mindfulness and tDCS, all participants, regardless of the allocated group, reported having received active tDCS.

All participants were female, attended formal school for over 8 years, had no associated comorbidities, and there were no reports of smoking or alcoholism. The main demographic and clinical data of the baseline are summarized in Table 1. No statistically significant differences were found between the groups at baseline.

**Table 1:** Demographic and clinical characteristics of the participants at baseline.

**Variables**	**Sham group (*n* = 14)**	**Active group (*n* = 16)**	***p*-value**
Age, years, M ± SD	33.06 (11.01)	32.75 (8.90)	n.s
Gender, female, *n*	14	16	n.s
Schooling>8 years, *n*	14	16	n.s
Smoking/ alcoholism, *n*	0	0	n.s
Duration of chronic phase, years, M ± SD	11.28 (2.33)	11.56 (2.75)	n.s
Pain intensity, VAS score, M ± SD	8.07 (2.6)	8.4 (1.09)	n.s
Pain medication consumption >3x/week, *n*, %	9 (64)	10 (62.5)	n.s

*n, number of participants; M, average in years; SD, standard deviation; %, percentage frequency; p, significance; n.s, not significant (p > 0.05); VAS, visual analogue scale*.

Regarding the safety of the procedure used, both groups presented an equivalent amount of adverse effects, all cases considered of mild intensity, having some difference in their clinical presentation (Table 2), with these effects being consistent with the literature on the subject (Vecchio et al., 2016; Andrade et al., 2017). The predominant report in both the sham current group and the active current group was sleepiness, with 40% more frequent in the sham current group. No participants had any serious side effects during the study.

**Table 2:** Distributed adverse effects on active tDCS and sham tDCS groups associated with mindfulness.

**Adverse effects**	**Active tDCS and mindfulness**	**Sham tDCS and mindfulness**
Tingling, *n*, %	2 (14.3)	0
Mild headache, *n*, %	0	2 (14.3)
Skin reaction, *n*, %	2 (14.3)	0
Sleepiness, *n*, %	3 (21.4)	5 (35.7)

*n, number of participants; %, percentage frequency*.

### 3.2. Primary Outcome

The intergroup comparison regarding the full attention level evaluation of participants (FFMQ-BR questionnaire) did not obtain significant results at both baseline (*U* = 98.5, *p* = 0.29) or post-intervention (*U* = 94.0, *p* = 0.23). Significant results were obtained in both groups regarding the intragroup evaluation, active tDCS (*t* = −3.0, *p* = 0.00, *r* = 0.75) and sham tDCS (*t* = −1.9, *p* = 0.03, *r* = 0.49), with the greatest effect on the active current group.

### 3.3. Secondary Outcome

With respect to the inability to perform daily living activities due to migraine (MIDAS questionnaire), no statistically significant difference was found between groups at baseline (*U* = 106.0, *p* = 0.41) or at the endpoint (*U* = 104.5, *p* = 0.38; Table 3). Significant results were observed in both groups in the intragroup comparison, active current group (*T* = − 2.1, *p* = 0.02, *r* = 0.53) and sham current group (*T* = − 1.8, *p* = 0.04, *r* = 0.49), with a larger effect size in the active current group (Table 4).

**Table 3:** Intergroup comparison of the three clinical outcomes in pre and post treatment of active and sham conditions (active tDCS: *n* = 16, sham tDCS: *n* = 14).

	**Pre, M ± SD**	**Post, M ± SD**	**Pre, *U*, *p***	**Post, *U*, *p***
MIDAS	24.0 ± 9.4	18.0 ± 6.3	*U* = 106.0, *p* = 0.41	*U* =104.5, *p* = 0.38
HIT-6	64.0 ± 4.7	60.8 ± 3.8	*U* = 81.5, *p* = 0.10	*U* =57.5, *p* = 0.01
FFMQ-BR	2.6 ± 0.5	2.9 ± 0.6	*U* = 98.5, *p* = 0.29	*U* =94.0, *p* = 0.23

*M, mean; SD, standard deviation; U, Mann-Whitney test; p, one-sided significance level*.

**Table 4:** Active tDCS vs. sham tDCS intragroup comparison associated with mindfulness of the three clinical outcomes in pre and post treatment.

	**Pre active tDCS, M ± DP**	**Pre sham tDCS, M ± DP**	**Post active tDCS, M ± DP**	**Post sham tDCS, M ± DP**	**Active tDCS, T, p, r**	**Sham tDCS, *T*, *p*, *r***
MIDAS	23.9 ± 8.1	24.3 ± 11.0	18.0 ± 7.1	18.0 ± 5.6	*T* = 2.1, *p* = 0.02, *r* = 0.53	*T* = -1.8, *p* = 0.04, *r* = 0.49
HIT-6	65.2 ± 4.1	62.6 ± 5.1	60.9 ± 3.4	59.6 ± 2.9	*T* = −2.9, *p* = 0.00, *r* = 0.71	*T* = −2.0, *p* = 0.02, *r* = 0.54
FFMQ-BR	2.6 ± 0.5	2.5 ± 0.5	3.0 ± 0.5	2.8 ± 0.6	*T* = −3.0, *p* = 0.00, *r* = 0.75	*T* = −1.9, *p* = 0.03, *r* = 0.49

*M, mean; SD, standard deviation; T, Wilcoxon station test; p, one-sided significance level; r, effect size*.

**Figure 4:**
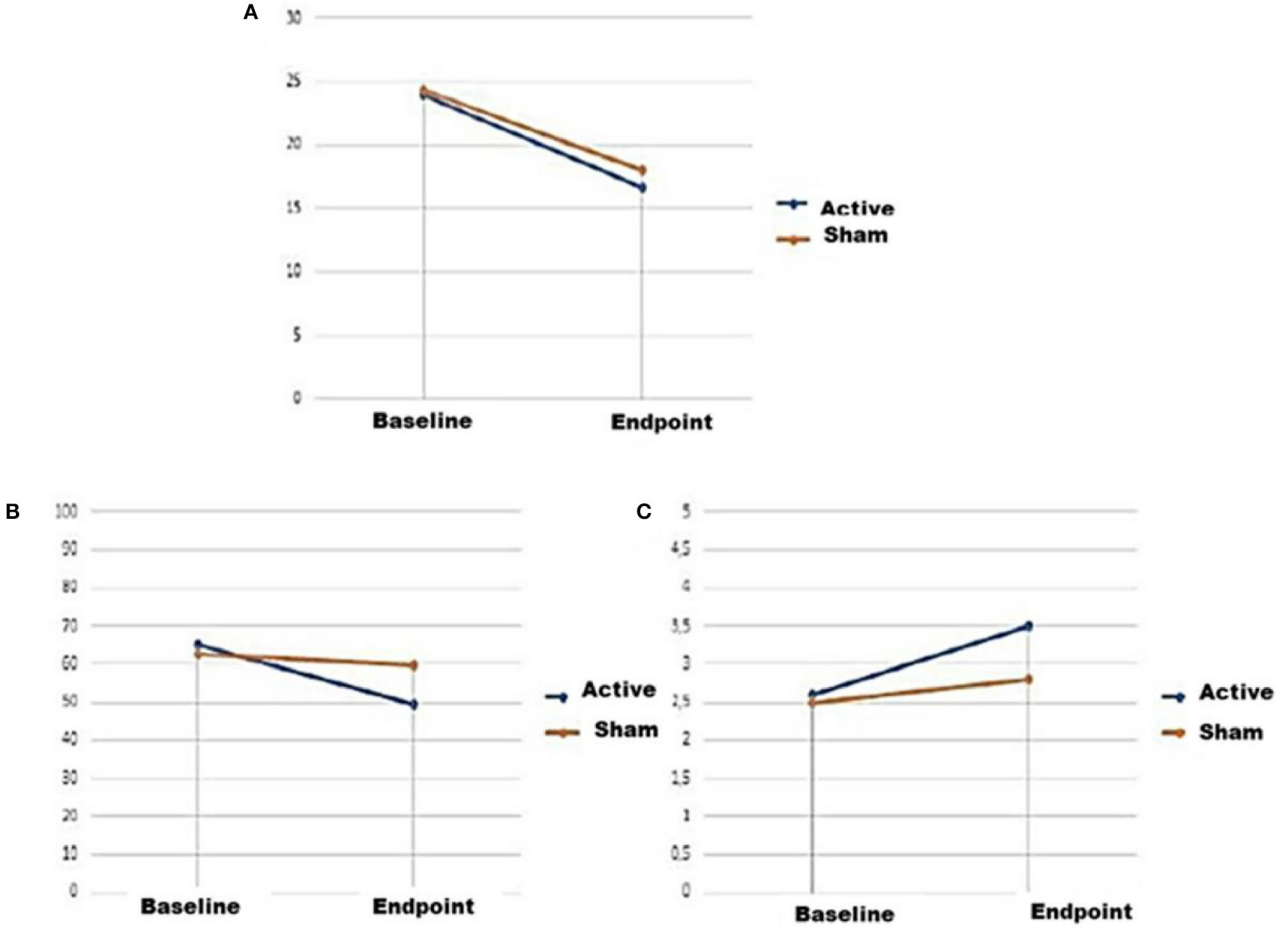
Line graphs representing mean ± mean standard error of the three clinical outcomes at baseline and endpoint of active and sham conditions. **(A)** Scores on the MIDAS questionnaire, **(B)** scores on the HIT-6 questionnaire, **(C)** scores on the FFMQ-BR questionnaire.

**Figure 5:**
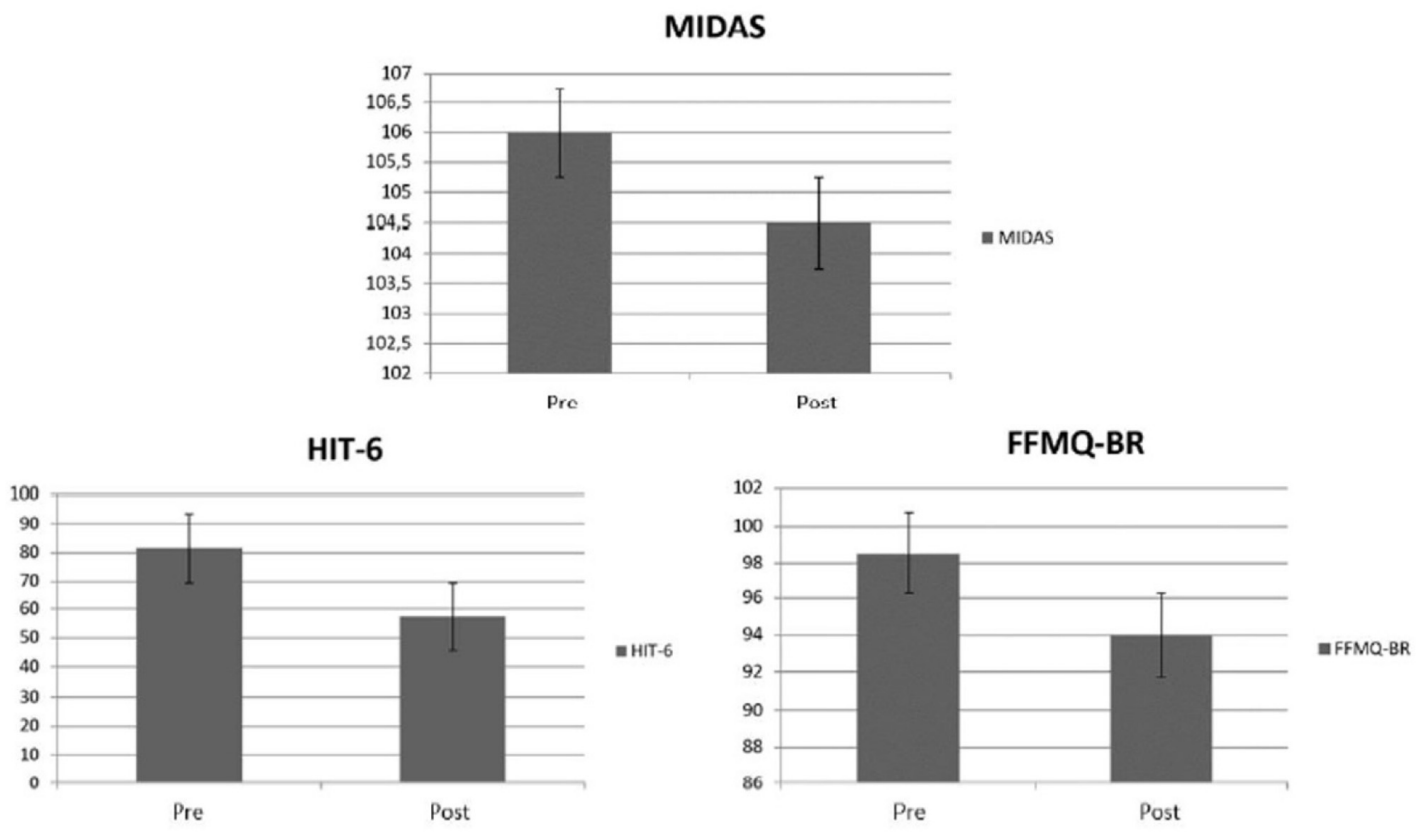
Bar graphs representing the Man-Whitney Test scores ± standard error of the three clinical outcomes at baseline and endpoint of active and sham conditions. Questionnaires scores, respectively, MIDAS, HIT-6, and FFMQ-BR.

The intergroup analysis regarding the impact of headache on the participants' quality of life (HIT-6 questionnaire) did not result in a significant comparison of the baseline (*U* = 81.5, *p* = 0.10), but it did at the end of treatment (*U* = 57.5, *p* = 0.01, *r* = 0.42). The intragroup evaluation was statistically significant for both the active current group (*T* = − 2.9, *p* = 0.00, *r* = 0.71) and the sham current group (*T* = − 2.0, *p* = 0.02, *r* = 0.54), presenting a larger effect size for the active tDCS, similar to the other outcomes. The comparisons between the outcomes are explained in Figures 4, 5.

## 4. Discussion

In this study, it was observed that the active current group showed an increase in the level of full attention and improvements in the inability to perform activities of daily living and in the impact of the participants' headache. The sham group was also benefited, but with smaller effect sizes than the active group. However, only the inability to perform activities of daily living showed significant differences in the comparison between both groups in the post-treatment.

Since both groups performed mindfulness practices, this would be a possible explanation for the improvement in the results of all outcomes in the sham group. The use of mindfulness as a therapeutic practice has been shown in previous studies to be safe and viable in adults with chronic migraine (Wells et al., 2014), demonstrating to reduce functional disability and suffering related to migraine (Smitherman et al., 2015), promoting greater awareness, de-identification with self, greater optimism related to sensations and feelings (Feuille and Pargament, 2015), reducing relapses by minimizing triggers that trigger crises such as anxiety and tension, increasing resilience to stress and the effective regulation of heart rate (Azam et al., 2016), in addition to improving the perception of pain intensity and quality (Bakhshani et al., 2016). Another justification would be the performance of the placebo effect, which according to the definition found in the literature is “the favorable result that derives from the patient's positive expectations and not from the physiological mechanism of the treatment itself” (Teixeira, 2008).

This study proved to be favorable to the hypothesis that mindfulness practice and tDCS in the left DLPFC can be combined for synergistic effects in reducing pain symptoms and in expanding the level of full attention of chronic migraineurs, since the groups with active current showed better results than simulated current. According to previous studies, such occurrence stems from the neuroplasticity provided by tDCS, which increases the brain's ability to reorganize in response to other clinical interventions (Ahn et al., 2019), as well as facilitating neural activity patterns in progress (Hunter et al., 2018). According to previous studies, mindfulness and tDCS seem to having complementary mechanisms of action enabling to minimize rumination, which is a relevant causal factor for depression and also a potential trigger for migraine attacks (Azam et al., 2016; Monnart et al., 2019). A clinical trial associating mindfulness with tDCS in healthy individuals concluded that meditation sessions are positively reinforced (in quality of mindfulness levels) with the use of this neuromodulation tool (Badran et al., 2017). However, there is a study with alcohol patients undergoing treatment which found no evidence of synergism between mindfulness and tDCS with regard to improving the symptoms of this clinical condition (Witkiewitz et al., 2019).

All participants in the present study were female. It was initially thought to select representatives of both genders, however, as the demand for volunteers was massive among women and because the pathology under evaluation is predominant in this gender (Domingues et al., 2009; Queiroz et al., 2009; Cauás et al., 2010), we chose to select only female participants. The influence of female hormonal factors is considered to be potentially relevant in the pathophysiology of migraine (Parra et al., 2015) and this exclusive female selection can be considered a limiting factor, since it is not known whether the results of tDCS therapy could differ in males.

In this study, we did not exclude patients who were receiving previous pain control medication (only analgesics and/or non-steroidal anti-inflammatory drugs) and we did not perform specific analyzes with regard to the quantity and type of drug in each of the groups as outcome predictors, which is therefore a limitation. However, we selected participants using stable doses of pain medications and an approximate amount between them for at least 2 months before the intervention. We also asked them not to modify this use during the treatment period.

Adherence to home practices of mindfulness was monitored by filling in individual tables with the schedule performed and if there was an interruption with a later restart, as previously instructed. However, some participants claimed to sporadically forget filling in some exercises (maximum of three annotation gaps in the table of four participants), despite having informed that they performed the practice on the non-registration days. Four participants reported difficulty in home training (deconcentrating, not finding an adequate place to listen to the audios at home or not identifying with the tool), with three dropouts from the study due to inadequacy to exercise mindfulness. The absence of notes in the table provided can be considered a limitation, since it makes it difficult to assess adherence to this activity to complement the intervention associated with tDCS.

Some methodological aspects can also be considered limiting, such as the small size of the groups, which can decrease the statistical power to detect minor effects. In addition, the subjects were recruited from a service specialized within a tertiary institution, so they may not be representative of the general chronic migraine population. Another point would be the limitations of tDCS power found in the literature in relation to intra and interindividual variables (Boros et al., 2008; Cunningham et al., 2015). Thus, strategies were adopted in order to systematize the process in each stimulation session, such as standardization of the electrode size and the use of coordinates to locate the target region (left DLPFC).

Although the proportion of adverse effects was equivalent in both groups (Table 2) and this occurrence was compatible with that already mentioned in similar literature (Vecchio et al., 2016; Andrade et al., 2017), there was 40% more drowsiness in the sham current group. We speculate that the synergism between mindfulness and tDCS in the active stimulation group minimized the sleepiness of the full current group, since the literature demonstrates that the combination of cognitive training with brain stimulation seems to be conducive to the current neural activity patterns associated with control and regulation of attention (Clark and Parasuraman, 2014; Hunter et al., 2018).

It is indicated that future studies evaluate other stimulation parameters, such as intensity and polarity of the stimulation. Therefore, computational models (meaning models which predict current flow in the target areas) can be useful to refine the design of future studies, thus optimizing the effects of stimulation and providing specific information about the inter-individual differences that may influence tDCS effects on migraine. The inclusion of three more arms in the study (one with active tDCS only, another with tDCS sham only, and another with only mindfulness practice) can be useful to provide greater reliability in assessing the synergism of combination therapy and in more accurate conclusions about the placebo effect.

Despite the limitations discussed, the results of this study provide the first therapeutic evidence of the practice of mindfulness associated with anodic tDCS of the left DLPFC with its consequent increase in the level of full attention and its analgesic benefits in the clinical symptoms of patients with chronic migraine. It is hoped that this study will encourage further research on the interaction between tDCS and mindfulness training to reduce the negative effects of chronic migraine, including EEG recording, assessment of anxiety and sleep quality.

## Data Availability Statement

The original contributions presented in the study are included in the article/supplementary materials, further inquiries can be directed to the corresponding author/s.

## Ethics Statement

The studies involving human participants were reviewed and approved by Comitê de Ética em Pesquisa—Centro de Ciências da Saúde—Federal of University Paraíba. The patients/participants provided their written informed consent to participate in this study.

## Author Contributions

SA and LP have designed the study and managed all stages of the design and writing process. LP has performed the data analysis and has written the first draft of the manuscript. LL has contributed to the interpretation and review of the data. EA, PB, JS, and JF have operationalized tDCS. LH and AL have written the last draft of the manuscript. All authors have contributed to the revision of the manuscript, read, and approved the submitted version.

## Funding

This study was financed in part by the Coordination for the Improvement of Higher Education Personnel—Brazil (CAPES)—Finance Code 001.

## Conflict of Interest

The authors declare that the research was conducted in the absence of any commercial or financial relationships that could be construed as a potential conflict of interest.

## Publisher's Note

All claims expressed in this article are solely those of the authors and do not necessarily represent those of their affiliated organizations, or those of the publisher, the editors and the reviewers. Any product that may be evaluated in this article, or claim that may be made by its manufacturer, is not guaranteed or endorsed by the publisher.
